# *Musa* sp. Leaves Extract Ameliorates the Hepato-Renal Toxicities Induced by Cadmium in Mice

**DOI:** 10.3390/molecules27020559

**Published:** 2022-01-16

**Authors:** Karim Samy El-Said, Shaimaa Hussein, Barakat M. Alrashdi, Heba A. Mahmoud, Mahrous A. Ibrahim, Mohamed Elbakry, Hala El-Tantawy, Doaa Ibrahim Kabil, Sabry A. El-Naggar

**Affiliations:** 1 Biochemistry Division, Chemistry Department, Faculty of Science, Tanta University, Tanta 31527, Egypt; mohamed.elbakry@science.tanta.edu.eg; 2 Pharmacology Department, College of Pharmacy, Jouf University, Sakaka 41412, Saudi Arabia; sshassan@ju.edu.sa; 3 Biology Department, College of Science, Jouf University, Sakaka 41412, Saudi Arabia; bmalrashdi@ju.edu.sa; 4 Pharmacology Department, Faculty of Medicine, Tanta University, Tanta 31527, Egypt; heba.mahmoud@med.tanta.edu.eg; 5 Forensic Medicine and Clinical Toxicology, College of Medicine, Jouf University, Sakaka 41412, Saudi Arabia; mabdulbaset@ju.edu.sa or; 6 Forensic Medicine and Clinical Toxicology Department, Faculty of Medicine, Suez Canal University, Ismailia 41522, Egypt; 7 Zoology Department, Faculty of Science, Ain Shams University, Cairo 11566, Egypt; halaeltantawi@hotmail.com; 8 Nutrition and Food Science, Faculty of Specific Education, Tanta University, Tanta 31527, Egypt; doaa.kabeal@sed.tanta.edu.eg; 9 Zoology Department, Faculty of Science, Tanta University, Tanta 31527, Egypt

**Keywords:** *Musa* sp., phytochemicals, antioxidants, cadmium, toxicity

## Abstract

Heavy metals intoxication causes several health problems that necessitate finding new protective and therapeutic approaches. This study aimed to evaluate the impact of *Musa* sp. leaves extract (MLE) on hepato-renal toxicities induced by cadmium (Cd) in male mice. The phytochemical screening, metal chelating activity (MCA), and the median lethal dose (LD_50_) of MLE were determined. Fifty CD-1 male mice were used and intraperitoneally (i.p.) injected with MLE (1000 to 5000 mg/kg b.wt) for MLE LD_50_ determination. Another 50 mice were used for evaluating the effect of MLE on Cd toxicity. Blood samples were collected for hematological, liver, and kidney functions assessments. Liver tissue homogenates were used for determination of oxidant/antioxidant parameters. Liver and kidney tissues were harvested for histopathological and molecular investigations. MLE showed potent in vitro antioxidant activities. The MCA and LD_50_ of the MLE were 75 µg/mL and 3000 mg/kg b.wt, respectively. MLE showed beneficial therapeutic activity against hepato-renal toxicities in Cd-intoxicated mice, evidenced by improving the hematological, biochemical, histopathological, and molecular alterations.

## 1. Introduction

Industrial, agricultural, transportation, and other daily activities all over the world increased the levels of pollution, particularly heavy metals in soil, water, and air environments [1,2]. The presence of heavy metals due to pollution led to harmful impacts on human health and the eco-system [3]. Acute and chronic toxicities due to heavy metals exposure led to irreversible damage in human and animal tissues and could induce cancer [4]. Furthermore, the accumulated heavy metals in the most vital tissues and organs—for instance, in the kidneys, liver, and bones—produce several health problems such as renal and hepatic dysfunction and bone diseases. A previous study reported that Cd toxicity promotes pathological changes through free radical initiation mechanisms [5].

The Food and Drug Administration (FDA) approved chelation therapy for removing the heavy metals from the body. In this regard, ethylene diamine tetra-acetic acid (EDTA) was used as a synthetic chelating agent to remove heavy metals from the body; thus, it reduces inflammation and tissue damage [6]. A previous study showed that medicinal herbs can be potentially used in the treatment of the heavy metals poisoning. For instance, different tomato extracts have been shown to clear the bioaccumulation of heavy metals in rats [7].

Phytochemical constitutes were reported to protect against heavy metals toxicity in rats, and treatment with some medicinal plants ameliorated the heavy metal toxicity in experimental animals [8,9,10]. Banana (*Musa* sp.) is an herbaceous flowering plant that grows worldwide. In a sub-chronic toxicity study of banana extracts, no mortality or biochemical alterations in adult male albino mice were noticed [11]. The biological and biomedical applications of *Musa* sp. have been investigated, and *M. paradisiaca* showed antiulcer activity in rats [12]. Gel from unripe banana peel caused better epithelization of wounds healed in Wistar rats in addition to its anti-inflammatory and antioxidant potential [13]. Ethanolic extracts of unripe bananas *M. sapientum* showed a high antimicrobial activity against micro-organisms and showed anti-ulcerogenic activity. *M. sapientum* flowers extract showed hypoglycemic activity, improvement in glucose tolerance, and antioxidant activity in diabetics [14]. *M. sapientum* stem aqueous extract showed hepatoprotective activity against carbon tetrachloride-induced hepatotoxicity in rats [15]. *M. paradisiaca* stems extracts showed hematopoietic and immunomodulatory properties due to the stimulation and formation of erythropoietin by its phytochemicals [16]. Previous studies evaluated the biomedical activities of different parts of *Musa* sp.; *Musa* leaves showed the most potent antioxidant and biomedical activities [12,13,14,15,16,17]. Therefore, the present study aimed to investigate the impact of *Musa* leaves extract (MLE) against cadmium toxicity in albino mice.

## 2. Results

### 2.1. Phytochemical’s Analysis and Metal Chelating Activity of MLE

**Figure 1:**
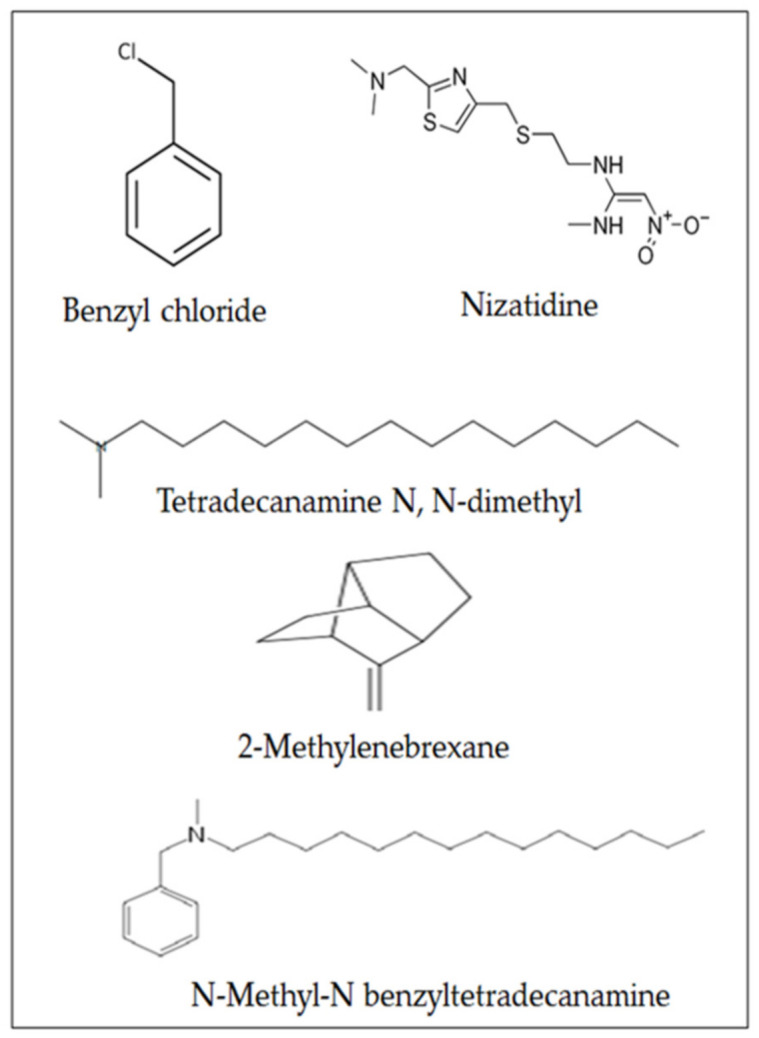
The most abundant phytochemical constituents of MLE.

The total phenolic, flavonoids, saponin, and anthocyanin content in the MLE were 2.92, 1.89, 0.297, and 0.469 mg/mL, respectively. In MLE, the total antioxidant capacity (TAC) was 0.162 mg/mL, while the DPPH radical scavenging was 71%, and the IC_50_ value was 0.685 mg/mL (Table 1). The metal chelating activity against ferrous ion showed that the IC_50_ of the extract for chelating activity was 75 µg/mL and was higher than the standard EDTA (IC_50_ = 20 µg/mL) (Table 1). Gas chromatography-mass spectroscopy (GC-MS) analysis showed that the most abundant phytochemical constitutes in MLE were benzyl chloride (PA: 6.46%), nizatidine (PA: 43.23%), 1-tetradecanamine *N*,*N*-dimethyl- (PA: 19.32%), 2-methylenebrexane (PA: 15.38%), and finally *N*-methyl-*N* benzyltetradecanamine (PA: 2.7%) (Table 2 and Figure 1).

### 2.2. The Median Lethal Dose (LD_50_) of MLE

After 24 h of MLE injection (i.p.), the LD_50_ was 3000 mg/kg b.wt; this result was obtained from two separate experiments (Table 3).

### 2.3. Treatment with MLE Decreased Cd Toxicity on Hematological Parameters

Cd injection led to significant decrease (*p* < 0.05) in both the total RBCs counts and Hb levels, while treatment with MLE returned these levels close to the normal values. In Cd-intoxicated mice, (Gp3), the total of WBCs and platelets counts were increased; however, the treatment with MLE restored these levels close to normal. Cd injection (Gp3) led to an increase in the percentages of neutrophils (%) and monocytes (%), while decreasing the percentage of lymphocytes (%). Treatment with MLE along with Cd-injection led to modulation of the percentage of the different types of WBCs (Table 4).

### 2.4. MLE Treatment Restores the Liver and Kidney Functions and Returns the Antioxidant Enzymes Close to Their Normal Levels

**Figure 2:**
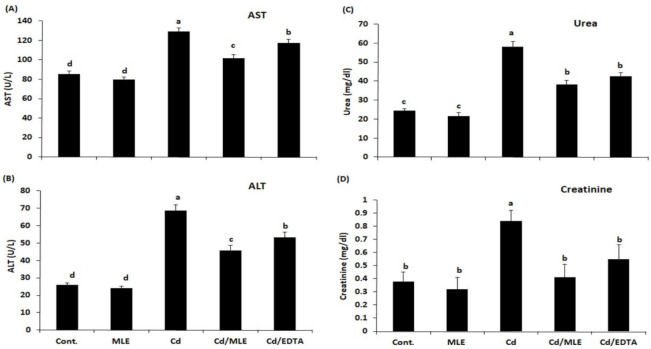
Serum AST, ALT, urea, and creatinine parameters in the different groups under study. (**A**) AST activity, (**B**) ALT activity, (**C**,**D**) urea, and creatinine levels. Tukey pairwise comparison; means that do not share a letter are significantly different.

**Figure 3:**
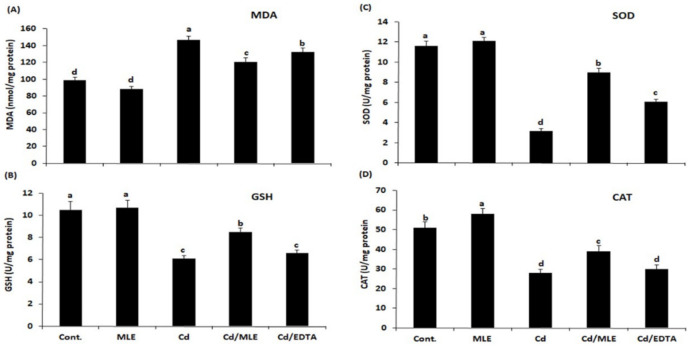
Hepatic oxidative stress parameters in the different groups under study. (**A**) Malondialdehyde (MDA), (**B**) reduced glutathione (GSH), (**C**,**D**) superoxide dismutase (SOD), and catalase (CAT) activities. Tukey pairwise comparison; means that do not share a letter are significantly different.

As compared with the control group (Gp1), Cd injection led to a significant increase (*p* < 0.05) in the levels of AST, ALT, urea, creatinine, and MDA (Figure 2 and Figure 3A). Concomitant treatment with MLE decreased the levels of the above parameters significantly (*p* ≤ 0.05). The levels of GSH, SOD, and CAT were decreased after the Cd-injection and were restored to their normal level after the co-treatment with MLE when compared with Cd/EDTA-treated mice (Figure 3B–D).

### 2.5. MLE Ameliorated Cd-Induced Liver and Kidney Tissue Damage

**Figure 4:**
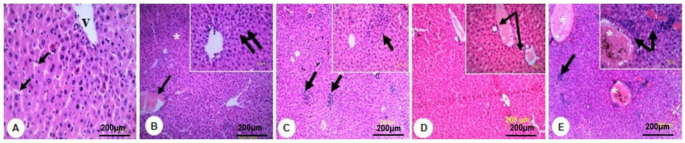
Photomicrographs of liver sections of rats stained with hematoxylin and eosin (H&E) (100×, 200×). (**A**) Control group showing central vein (v), cords of polyhedral hepatocytes, and blood sinusoids (arrows). (**B**) MLE-treated group showing the normal lobular hepatic architecture with slight dilation and congestion of some central veins (arrows) and blood sinusoids. (**C**) Cd-treated group showing clumps of polymorphonuclear neutrophils mixed with lymphocytes infiltrates (arrow) and necrotic hepatocytes. (**D**) Cd/MLE group showing a few clumps of polymorphonuclear cells and lymphocytes infiltrates in the hepatic parenchyma (double arrows), some necrotic hepatocytes in degenerated areas (asterisks), and congestion of dilated central veins (arrow). (**E**) Cd/EDTA group showing no lobular hepatic architecture, extensive congestion of the central veins with cellular infiltration (asterisks), and small clumps of leukocytes and lymphocytic cells (arrows).

Microscopic examination of the normal liver sections in the control group showed defined hepatic lobules. Each lobule was formed of cords of hepatocytes radiating from the central veins (CV). The cells were separated by narrow blood sinusoids (BS) that were lined by Kupffer cells and endothelial cells. The hepatocytes were polyhedral with acidophilic cytoplasm and rounded darkly stained nuclei (Figure 4A). In the group of mice that was treated with MLE alone, typical lobular hepatic architecture with dilated and slight congested central vein was shown (Figure 4B). Necrotic areas infiltrated with clumps of polymorphonuclear neutrophils (PMN) mixed with lymphocytes and pyknotic hepatocytes were noticed in Cd-intoxicated group (Figure 4C). A few clumps of the PMN cells and lymphocytes infiltrated the hepatic parenchyma. Some necrotic hepatocytes in the degenerated areas of the tissue and less congested CV were exhibited in the tissues of mice treated with Cd and MLE (Figure 4D). The hepatic tissue lost, extensive congestion of the CV, inflammatory cells, and masses of leukocytes and lymphocytic cells was observed in the liver tissues of mice that were treated with Cd and EDTA (Figure 4E).

**Figure 5:**
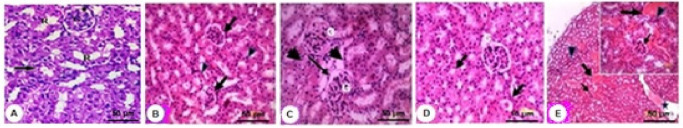
Photomicrographs of kidney sections of rats stained with hematoxylin and eosin (H&E) (200×, 400×). (**A**) Control group showing the renal cortex with the glomeruli (G) that was surrounded by Bowman’s capsule of simple squamous epithelial cells (thin arrow). The renal convoluted tubules are lined with simple cuboidal and simple squamous epithelial cells (thick arrows). (**B**) MLE-treated group showing normal tissue architecture with glomeruli (G) and renal tubules (arrows). (**C**) Cd-treated group showing shrinkage of the glomeruli (G). Renal tubular necrosis, highly eosinophilic epithelial cells (thick arrow), cytoplasmic vacuolated cells that lined the renal tubules (thin arrows), and cellular infiltration in the intertubular sites (circles). (**D**) Cd/MLE group showing slight shrinkage of glomeruli (arrows), well-developed simple cuboidal cells that lined the renal tubules (arrowheads), and no cellular infiltration in the tissue. (**E**) Cd/EDTA group showing severe congestion of the blood capillaries (thick arrows), bleeding in the main blood vessels (star), the malformed glomeruli (thin arrows), and the renal tubules lined with altered eosinophilic and hyperplasia of epithelial cells (arrowheads).

Histological alterations were recorded in the renal tissues of groups treated with Cd. Glomeruli shrinkage, tubular necrosis, and inflammatory cell infiltration were recorded. Highly eosinophilic cells and vacuolated cytoplasm were observed in the epithelial lining cells of the renal tubules (Figure 5C) compared with the normal kidney histological architecture (Figure 5A). The kidney histological architecture was almost typical in the groups that were treated with MLE (Figure 5B). The histological alterations induced by Cd were markedly reduced by the treatment with MLE, in which slight shrinkage of the glomeruli, no tubular necrosis, and no cellular infiltration were observed (Figure 5D). The severe congestion and the bleeding in the blood capillaries were the most noticeable features of the histological lesions in the tissues of groups treated with Cd/EDTA. Malformed glomeruli and hyperplasia of the tubular lining cells were revealed (Figure 5E).

### 2.6. Molecular Analysis of the Pro-Inflammatory Genes

The results show that in the Cd-intoxicated group, there was upregulation in the mRNA expression levels of the pro-inflammatory genes (TGF-β1, NFκ-β, and COX-1) in the liver and kidney tissues when compared with their controls. Meanwhile, the expression levels of these genes were downregulated in the liver and kidney tissues of mice treated with MLE post Cd intoxication when compared with the naïve control group and group treated with Cd/EDTA (Table 5).

## 3. Discussion

*Musa* sp. has been reported to have several therapeutic benefits, including antioxidant, anti-diabetic, anti-cancer, and anti-inflammatory activities [18]. The current study evaluated the effect of MLE as a natural chelating agent against heavy metal-intoxicated mice. A phytochemical analysis showed that MLE contains adequate levels of phenolics and flavonoids. The presence of these secondary metabolites in MLE may provide pharmacological and biochemical actions upon its administration to animals [16,17]. These findings agreed with a report that indicated the presence of phytochemicals in *M. paradisiaca* plant [16]. GC-MS analysis of MLE showed several bioactive compounds. The most abundant phytochemical constitutes in MLE were benzyl chloride, nizatidine, tetradecanamine *N*,*N*-dimethyl, 2-methylenebrexane, and *N*-methyl-*N* benzyltetradecanamine. Benzyl chloride is used as a chemical intermediate in the manufacture of several industrial and pharmaceutical products [19]. Nizatidine is a histamine receptor antagonist that can be used in the treatment of allergic disease [20]. Nizatidine, tetradecanamine *N*,*N*-dimethyl, and *N*-methyl-*N* benzyltetradecanamine are bioactive compounds that are found in several medicinal plants, such as *Citrullus colocynthis* and *Commiphora myrrh* [21,22].

The results report that the IC_50_ of metal chelating activity of MLE was 75 µg/mL; this postulates that MLE has a potent chelating capacity in vitro and that this could explain the role of MLE in heavy metals detoxification in vivo. Our data were consistent with a previous study that reported the IC_50_ of chelating effect on heavy metals [23]. Upon injection with MLE, up to 3000 mg/kg did not show acute toxicity or mortality, and this finding showed that MLE was safe for administration in animals. This finding was consistent with a study reported that there was no acute toxicity of MLE up to 2000 mg/kg [13].

The data show that Cd injection in mice caused significant alterations in hematological parameters, including RBCs, WBCs, platelets, and Hb. Cd injection, furthermore, increased the percentages of neutrophils (%) and monocytes (%), while decreasing the percentage of lymphocytes (%). These results agreed with a previous study that demonstrated that Cd injection caused anemia, thrombocytosis, and decrease in lymphocytes in experimental animals [24,25]. It has been reported that Cd injection into mice increased myeloid and monocytic cells in bone marrow [26]. The neutrophilia with leukocytosis observed in this study after-Cd injection could be due to the release and mobilization of neutrophils [27]. Treatment with MLE, along with Cd-injection, improved the alterations in the hematological parameters, evidenced by a return of RBCs count close to their normal level and restoration of the count of WBCs and platelets. Our results are in alignment with a previous study reporting that *M. paradisiaca* stem extract improved the hematological indices in rats by increasing erythropoietin, which in turn stimulated RBCs regeneration [28]. Ramu et al. (2017) investigated the protective effect of *M. paradisiaca* against free radical-induced damage in erythrocytes by phytosterols [29]. This was correlated with hepatic tissue damage and increased liver enzymes due to accumulation of Cd in hepatic tissues, which resulted in accumulation of lipid peroxides. The present study reported a significant increase in the activities of liver transaminases (AST and ALT), urea, and creatinine levels. In alignment with our results, it was reported that Cd injection increased urea and creatinine levels in rats [23]. Administration of MLE with Cd injection decreased the levels of the above parameters significantly. A previous study showed that *M. paradisiaca* improved kidney function in mice due to its phytochemical constituents [30].

In heavy metal-intoxicated mice, lipid peroxidation was increased, as indicated by the increase in MDA level, and oxidative stress was also increased, as indicated by decreased GSH, SOD, and CAT in liver tissues, which was in accordance with a previous report of Alhazzi (2008) [31]. Treatment with MLE reduced lipid peroxidation and oxidative stress, as indicated in this study by significantly decreased MDA levels and increased GSH, SOD, and CAT activity. MLE ameliorated the deleterious effects of Cd on liver tissues more than EDTA. In accordance with our results, previous experimental studies have confirmed the therapeutic potential and antioxidant activity of the *Musa* sp. extract. Lipid peroxidation decreased, and SOD increased upon treatment with *M. sapientum* root extracts in the experimental models [32]. MLE extract increased the GSH and SOD level, accompanied by a decrease in MDA, and demonstrated a hepatoprotective effect due to its antioxidant activity [15]. Furthermore, the efficacy of MLE extract and possible mechanism of anti-urolithiasis and antioxidant efficacy were evaluated in rat [33].

Histologically, the results indicate that Cd injection resulted in severe oxidative damage in the liver tissues, which was evidenced by the appearance of necrotic alterations, along with inflammatory cell infiltration. This result agreed with the previous study that reported the hazards of Cd on the liver [34]. Previous studies have suggested that Cd generates reactive oxygen species (ROS), causes oxidative damage to membrane lipids, disturbs membranes integrity, and involves cytotoxic and inflammatory mediators in the liver [4,35]. Production of ROS could be attributed to the direct actions of Cd on peroxidation [36]. Primary injury of cells resulting from binding of Cd to sulfhydryl groups in mitochondria and secondary damage initiated by the activation of Kupffer cells have been mentioned as possible mechanisms of the toxic effect of Cd on the liver. Moreover, Cd forms covalent and ionic bonds with atoms of sulfur, oxygen, and hydrogen present in the cellular components, causing significant homeostasis disruption [37].

The current work demonstrated histopathological changes in tubular cell necrosis in the kidney after Cd injection. This finding was confirmed by a previous report demonstrating that Cd induced nephrotoxicity in mice [38]. Cellular damage caused by Cd can be limited by free radical scavengers, which further supports the hypothesis that free radicals play an essential role in Cd toxicity. Treatment with EDTA has not resulted in improvement in the hepatic and renal architecture. Extensive congestion and severe bleeding were shown in the liver and kidney tissues. MLE showed a potential therapeutic effect on the hepatic and renal tissues against the toxicity of the Cd. The degeneration and the necrotic signs were less recorded, and the degenerative features were reduced after treatment with MLE. A similar finding was reported post paracetamol administration in mice and treated with *M. sapientum* [39]. It was reported that *M. sapientum* has effective antimutagenic activities because of its content of flavonoids and coumarins that could result in the antitoxic effect [40]. This study showed that Cd intoxication in mice led to overexpression in proinflammatory genes expression (TGF-β1, NFκ-β, and COX-1) in the liver and kidney tissues. Treatment with MLE after Cd injection in mice improved the inflammatory condition caused by Cd in the liver and kidney tissues more than EDTA, which was evidenced by downregulation of the previous proinflammatory genes. These findings agreed with a previous report that demonstrated the anti-inflammatory activity of MLE [18].

## 4. Materials and Methods

### 4.1. Materials

Cadmium chloride (CdCl_2_) and Na_2_EDTA were purchased from Merck Company (Darmstadt, Germany). Aspartate aminotransferase (AST), alanine aminotransferase (ALT), urea, creatinine, superoxide dismutase (SOD), catalase (CAT), and malondialdehyde (MDA) kits were purchased from Bio-Diagnostic Company (Cairo, Egypt). The other chemicals were purchased from the local chemical trade companies.

### 4.2. Preparation of MLE

*Musa* sp. leaves (MLE) were collected from a banana farm in El-Gharbiah governorate, Egypt, then transferred into the laboratory. The plant materials were identified and authenticated by taxonomist in the Botany Department, Faculty of Science, Tanta University. Leaves were washed twice to eliminate any chemicals and dust materials, then were cut into very fine pieces and left to dry in shade. Then grinding in a mechanical mortar and 50 g of the powder were mixed vigorously with 500 mL 70% (*v*/*v*) ethanol. The hydro-alcoholic extracts were filtered, the solvent was air-dried and concentrated in a vacuum evaporator, then the extracts were weighed, suspended in 0.9% sterile saline, and stored at −20 °C for further use.

### 4.3. Phytochemical’s Analysis of MLE

Total phenolic content of the extracts was determined using Folin–Ciocalteau reagent; the absorbance was determined at 730 nm using a spectrophotometer. The total phenolic content was expressed as milligrams (mg) gallic acid equivalents per gram of extracts using a calibration curve [41]. Total flavonoids were determined using the aluminum chloride colorimetric method, expressed as (mg) quercetin equivalent per gram of extract from a calibration curve of quercetin [42]. The phosphomolybednum method was used to determine the total antioxidant capacities that were expressed as ascorbic acid equivalent [43]. Free radical scavenging capacity was evaluated spectrophotometrically; the absorbance of sample (As) and control (Ac) was measured at 517 nm, the scavenging activity on the DPPH radical was expressed as inhibition percentage that equaled [(AC − AS)/(AC)] × 100 [44]. Saponin and anthocyanin contents were determined according to Ebrahimzadeh (1998) [45].

### 4.4. Gas Chromatography-Mass Spectrometry (GS-MS) Analysis of MLE

Phytochemical’s profile was determined in MLE by GC-MS analysis. The chemical composition of each sample was performed using a Trace GC 1310-ISQ mass spectrometer (Thermo Scientific, Austin, TX, USA) with a direct capillary column TG-5MS (30 m × 0.25 mm × 0.25 µm film thickness). The column oven temperature was initially held at 50 °C and then increased by 7 °C/min to 230 °C, held for 2 min, increased to the final temperature of 300 °C by 30 °C/min, held for 2 min. The injector and MS transfer line temperatures were kept at 270 and 260 °C, respectively. Helium was used as a carrier gas at a constant flow rate of 1 m/min. The solvent delay was 3 min, and diluted samples of 1 µL were injected automatically using an Auto Sampler AS1300 coupled with GC in split mode. EL mass spectra were collected at 70 eV ionization voltages over the range of *m*/*z* 45–600 in full scan mode. The ion source temperature was set at 200 °C. The components were identified by comparison of their retention times and mass spectra with those of WILEY 09 and NIST 11 mass spectral database.

### 4.5. Metal Chelating Activity (MCA) of MLE

The chelation power of ferrous ions by MLE was estimated, and the absorbance of the solution was measured at 562 nm. The percentage inhibition of ferrozine–Fe^2+^ complex formation was calculated as [(A0 − As)/Ast] × 100, where A0 was the control absorbance, and As/Ast was the extract/standard absorbance [46].

### 4.6. Mice

One hundred male Swiss albino mice (20 ± 2 g) were obtained from the National Research Center (NRC, Cairo, Egypt) to determine LD_50_ of MLE and assess its chelation activity. Animals were housed (5/cage) in 12 h/12 h dark/light cycle under laboratory conditions of temperature and humidity. Mice were kept for a week for adaptation before starting the experiment. The experimentation, transportation, and care of the animals were performed and handled in compliance with the ethical guidelines approved by the animal care and use committee, Faculty of Science, Tanta University (ACUC-SCI-TU-88), Egypt, and according to the National Institutes of Health Guide for the Care and Use of Laboratory Animals (NIH Publications No. 8023, revised 1996).

### 4.7. The Median Lethal Dose (LD_50_) of MLE

To determine the LD_50_ after intraperitoneal (i.p.) injection of MLE, 50 mice were divided into two main groups (*n* = 25), with each group further subdivided into 5 subgroups (5 mice of each). Then, the first and second subgroups were administered i.p. with different doses of MLE (1000–5000 mg/kg). Mice were then monitored for 24 h to determine the acute toxicity. The LD_50_ value was calculated according to the following equation:

LD_50_ = LDy − Σ (Dd × md)/N

where LDy = highest dose (LD_100_), N = number of animals per group, Dd = dose difference, Md = mean dead, LD_50_ = dose that killed 50% of test animals.

### 4.8. Experimental Design

Fifty mice were divided into five groups (10/each). The first group was used as a negative control. The second group was injected i.p. with MLE daily (300 mg/kg b.wt) which equaled one tenth of LD_50_. The third group was injected i.p. with Cd daily (5 mg/kg b.wt). The fourth group was injected with Cd and MLE in the same dose as in group 2 and 3, respectively. The fifth group was injected with Cd in the same dose and EDTA as a standard chelating agent (25 mg/kg b.wt). All treatments were continued for 10 consecutive days; at day 11, all mice were bled via the orbital plexus to collect blood for hematological assessments, and sera were separated for liver and kidney functions assessment. Liver tissues were prepared for oxidants and antioxidants parameters. Furthermore, liver and kidney sections were collected for histopathological investigations.

### 4.9. Hematological and Biochemical Assessments

Platelets, hemoglobin content (Hb g/dL), red blood cells (RBCs), white blood cells (WBCs), and differential counts were determined from fresh blood samples obtained from the orbital plexus of all groups under study using an electronic blood counter. Biochemical analyses were determined by using bio-diagnostic research kits as the follows: Serum alanine transaminases (ALT) (CAT. NO. AL 10 31), aspartate transaminases (AST) (CAT. NO. AS 10 61), urea (CAT. NO. UR 21 10), creatinine (CAT. No. CR 12 50), hepatic superoxide dismutase (SOD) (CAT. No. SD 25 21), catalase (CAT) (CAT. No. CA 25 17), malondialdehyde (MDA) (CAT. No. MD 25 29), and reduced glutathione (GSH) levels (CAT. No. GR 25 11).

### 4.10. Histopathological Investigation

Tissue specimens of liver and kidney were harvested and fixed in 10% formalin. Paraffin blocks were prepared after completing the tissue processing in different grades of alcohol and xylene. Sections (5 μm) were prepared from paraffin blocks using a microtome, stained with hematoxylin and eosin, which were observed under a light microscope (Optika, B-350, Ponteranica, Bergamo, Italy) to examine gross cellular damage.

### 4.11. Gene Expression Analysis

Real-time PCR with SYBR Green was used to measure mRNAs expression of tumor growth factor beta-1 (TGF-β1), nuclear factor kappa-beta (NFκ-β), and cyclooxygenase-1 (COX-1) genes in the liver and kidney tissues of all groups under study, with β-actin as an internal reference. The isolated cDNA was amplified using Maxima SYBR Green/ROX qPCR Master Mix following the manufacturer protocol (Thermo Scientific, Waltham, MA, USA, # K0221). The gene-specific primers for β-actin were ACCCACACTGTGCCCATCTA (forward) and CGTCACACTTCATGATG (reverse). TGFb-1 gene-specific primers were AAGAAGTCACCCGCGTGCTA and TGTGTGATGTCTTTGGTTTTGTCA for forward and reverse directions, respectively. The specific primers for NFκ-β were CCTAGCTTTCTCTGAACTGCAAA (forward) and GGGTCAGAGGCCAATAGAGA (reverse). The COX-1 primers were CCCAGAGTCATGAGTCGAAGGAG for forward and CAGGCGCATGAGTACTTCTCGG for reverse. The web-based tool (http://www-genome.wi.mit.edu/cgi-bin/primer/primer3_www.cgi, accessed on March 2021) was used to design these primers based on published sequences. Primer sequence similarity to other known sequences was checked with BLAST (www.ncbi.nlm.nih.gov/blast/Blast.cgi, accessed on March 2021).

### 4.12. Statistical Analysis

One-way analysis of variance (ANOVA) was used to assess the significant differences among treatment groups. Dunnett’s test was used to compare all groups against the control group to show the significant effect of treatment. The criterion for statistical significance was set at *p* ≤ 0.05. All data are presented as mean ± SD.

## 5. Conclusions

This study highlighted evidence for the promising ameliorative effect of MLE against hepato-renal toxicities induced by cadmium (Cd) in mice. The MLE showed potent antioxidant and metal chelating activities in vitro and in Cd-intoxicated mice. Furthermore, MLE improved hematological, biochemical, histopathological, and molecular changes induced by Cd in the liver and kidney tissues of mice.

## Figures and Tables

**Table 1:** Phytochemical analysis of *Musa* sp. leaves extract (MLE).

Phytochemical Parameters	MLE
Phenolics (mg/mL)	2.92
Flavonoids (mg/mL)	1.89
TAC (PMA) (mg/mL)	0.162
DPPH %	71
IC_50_ (mg/mL)	0.685
Saponin (mg/mL)	0.297
Anthocyanin (mg/mL)	0.469
Metal chelating activity (µg/mL)	75

**Table 2:** GC-MS analysis of *Musa* sp. leaves extract (MLE).

RT (min.)	Name	M. F.	M. Wt	Peak Area %
3.97	Benzyl chloride	C_7_H_7_Cl	126	6.46
9.89	1-Chlorooctadecane	C_18_H_37_Cl	288	0.78
10.11	4-Hydroxy-4-methyl-hex-5-enoic acid tert-butyl ester	C_11_H_20_O_3_	200	1.10
11.31	Decane, 1-chloro-	C_10_H_21_Cl	176	0.40
11.41	1-Dodecanol	C_12_H_26_O	186	1.37
11.98	Nizatidine	C_12_H_21_N_5_O_2_S_2_	331	43.23
13.75	Diethyl phthalate	C_12_H_14_O_4_	222	1.09
14.05	9-Octadecen-1-ol, (*Z*)-	C_18_H_36_O	268	0.45
15.20	Cholestan-3-ol, 2-methylene-,(3a,5a)-	C_28_H_48_O	400	0.56
15.27	1-Hexadecanol	C_16_H_34_O	242	0.73
15.76	1-Tetradecanamine, *N*,*N*-dimethyl-	C_16_H_35_N	241	19.32
18.13	Neophytadiene	C_20_H_38_	278	0.65
18.86	3,7,11,15-Tetramethyl-2-hexadecen-1-ol	C_20_H_40_O	296	0.48
19.61	Hexadecanoic acid, methyl ester	C_17_H_34_O_2_	270	0.70
20.29	*N*-Hexadecanoic acid	C_16_H_32_O_2_	256	1.40
22.38	9-Octadecenoic acid (Z)-, methyl ester	C_19_H_36_O_2_	296	0.71
22.57	2-Methylenebrexane	C_10_H_14_	134	15.38
23.03	Oleic Acid	C_18_H_34_O_2_	282	1.51
25.60	*N*-Methyl-*N*-benzyltetradecanamine	C_22_H_39_N	317	2.70
28.65	Di-*n*-octyl phthalate	C_24_H_38_O_4_	390	0.96

RT: Retention time; M. F.: Molecular formula; M. Wt: Molecular weight.

**Table 3:** Shows the calculations for LD_50_ determination of MLE after 24 h of intraperitoneal injection in mice.

Doses	1st exp.	2nd exp.	Md	Dd	Md × Dd
1 g/kg	0	0	0	0	0
2 g/kg	0	0	0	0	0
3 g/kg	2	2	2	1	2
4 g/kg	3	4	3.5	1	3.5
5 g/kg	5	4	4.5	1	4.5
Sum.					10

Md: mean death; Dd: dose difference.

**Table 4:** Complete blood count in the different groups of mice under the study.

Groups	RBCs (×10^6^/uL)	Hb (g/dL)	Platelets (×10^3^/uL)	WBCs (×10^3^/uL)	Differential Count		
					Neut. (%)	Lym. (%)	Mon. (%)
Naïve control	9.3 ± 0.9	11.7 ± 2.8	1.168 ± 49	8.3 ± 2.0	18 ± 1.5	80 ± 5.1	2.1 ± 0.9
MLE-treated	9.9 ± 0.8	12.8 ± 1.7	1.139 ± 42	8.1 ± 1.8	23 ± 1.8	72 ± 4.2	3.2 ± 1.0
Cd-treated	7.4 ± 1.3 ^ab^	13.9 ± 1.9 ^ab^	1.774 ± 51 ^ab^	12.5 ± 1.5 ^ab^	30 ± 2.8 ^ab^	40 ± 3.2 ^ab^	4.4 ± 1.5 ^ab^
Cd/MLE-treated	8.6 ± 1.2 ^c^	15.0 ± 2.4 ^c^	1.312 ± 45 ^c^	8.8 ± 2.3 ^c^	25 ± 3.8 ^c^	51 ± 2.7 ^abc^	3.5 ± 1.9 ^c^
Cd/EDTA-treated	7.9 ± 1.9	13.2 ± 2.6	1.014 ± 39 ^c^	9.1 ± 2.7	22 ± 3.1 ^c^	73 ± 2.5 ^c^	3.9 ± 1.9 ^c^

RBCs: red blood corpuscles; Hb: hemoglobin; WBCs: white blood cells. Neut: neutrophils; Lym: lymphocytes; Mon: monocytes. Means that do not share a letter are significantly different at *p* < 0.05.

**Table 5:** Fold changes of the mRNA expression of TGFβ-1, NFκ-β, and COX-1 genes in liver and kidney tissues of the different groups.

Tissue/Genes		Groups			
		Naïve Control	Cd-Treated	Cd/MLE-Treated	Cd/EDTA-Treated
Liver	TGFβ-1	1.04 ± 0.14	3.41 ± 0.29 ^ab^	2.11 ± 0.13	2.91 ± 0.15 ^ab^
	NFκ-β	1.09 ± 0.15	6.32 ± 0.54 ^ab^	2.65 ± 0.46 ^ab^	4.79 ± 0.26 ^ab^
	COX-1	1.05 ± 0.15	3.94 ± 0.25 ^ab^	1.82 ± 0.19	2.88 ± 0.24
Kidney	TGFβ-1	1.06 ± 0.16	2.67 ± 0.38	1.42 ± 0.16	2.08 ± 0.22
	NFκ-β	1.08 ± 0.14	4.47 ± 0.29 ^ab^	2.15 ± 0.34	3.19 ± 0.16 ^ab^
	COX-1	1.03 ± 0.16	3.75 ± 0.24 ^ab^	1.65 ± 0.15	2.68 ± 0.13

TGFβ-1: tumor growth factor beta-1; NFκ-β: nuclear factor kappa beta; COX-1: cyclooxygenase-1. Means that do not share a letter are significantly different at *p* < 0.05.

## Data Availability

Data sharing not applicable.

## Sample Availability

Samples are available from the authors.

## References

[1] Tchounwou P.B., Yedjou C.G., Patlolla A.K., Sutton D.J. (2012). Heavy metal toxicity and the environment. Molec. Clinic. Environ. Toxicol..

[2] Rehman M.U., Khan R., Khan A., Qamar W., Arafah A., Ahmad A., Ahmad A., Akhter R., Rinklebe J., Ahmad P. (2021). Fate of arsenic in living systems: Implications for sustainable and safe food chains. J. Hazard. Mater..

[3] Hubner R., Astin K.B., Herbert R.J. (2010). Heavy metal-time to move on from semantics to pragmatics. Environ. Monit..

[4] Kim H.S., Kim Y.J., Seo Y.R. (2015). An overview of carcinogenic heavy metal: Molecular toxicity mechanism and prevention. Cancer Preven..

[5] Liu J., Qu W., Kadiiska M.B. (2009). Role of oxidative stress in cadmium toxicity and carcinogenesis. Toxicol. Appl. Pharmacol..

[6] Shrihari J.S., Roy A., Prabhakaran D., Reddy K.S. (2006). Role of EDTA chelation therapy in cardiovascular diseases. Nation. Medic. J. India..

[7] Mehrandish R., Rahimian A., Shahriary A. (2019). Heavy metals detoxification: A review of herbal compounds for chelation therapy in heavy metals toxicity. J. Herbmed Pharmacol..

[8] Amin I., Hussain I., Rehman M.U., Mir B.A., Ganaie S.A., Ahmad S.B., Mir M.R., Shanaz S., Muzamil S., Arafah A. (2021). Zingerone prevents lead-induced toxicity in liver and kidney tissues by regulating the oxidative damage in Wistar rats. J. Food Biochem..

[9] Koubaa F.G., Chaâbane M., Turki M., Ayadi F.M., El Feki A. (2021). Antioxidant and hepatoprotective effects of Salvia officinalis essential oil against vanadium-induced oxidative stress and histological changes in the rat liver. Environ. Sci. Pollut. Res. Int..

[10] Dąbrowska Z., Dąbrowska E., Onopiuk B., Onopiuk P., Orywal K., Mroczko B., Pietruska M. (2019). The protective impact of black chokeberry fruit extract (*Aronia melanocarpa* L.) on the oxidoreductive system of the parotid gland of rats exposed to cadmium. Oxid. Med. Cell. Longev..

[11] Sumita B.S., Biswas M. (2013). Acute and sub-chronic toxicity study of *Musa paradisiaca* leaf extracts in mice. Advan. Pharm. Educ. Res..

[12] Ikpeazu O., Elekwa I., Ugbogu A., Arunsi U.O., Uche-Ikonne C. (2017). Preliminary evaluation of anti-ulcer potential of aqueous extract of fermented unripe *Musa paradisiaca* in Wistar rats. Am. J. Biomed. Res..

[13] Onasanwo S.A., Emikpe B.O., Ajah A.A., Elufioye T.O. (2013). Anti-ulcer and ulcer healing potentials of *Musa sapientum* peel extract in the laboratory rodents. Res. J. Pharmacog..

[14] Kappel V.D., Cazarolli L.H., Pereira D.F., Bárbara G., Postal B.G., Madoglio F.A., Reginatto F.H., Silva R.M.B. (2013). Beneficial effects of banana leaves (*Musa paradisiaca*) on glucose homeostasis: Multiple sites of action. Rev. Bras. Farmacogn..

[15] Dikshit P., Tyagi M.K., Shukla K., Sharma S., Gambhir J.K., Shukla R. (2011). Hepatoprotective effect of stem of *Musa sapientum* Linn in rats intoxicated with carbon tetrachloride. Ann. Hepatol..

[16] Imam M.Z., Akter S. (2011). *Musa paradisiaca* L. and *Musa sapientum* L.: A phytochemical and pharmacological review. J. Appl. Pharm. Sci..

[17] Karuppiah P., Mustaffa M. (2013). Antibacterial and antioxidant activities of Musa sp. leaf extracts against multidrug resistant clinical pathogens causing nosocomial infection. Asian Pac. J. Trop. Biomed..

[18] Sarma P.P., Gurumayum N., Verma A.K., Devi R. (2021). A pharmacological perspective of banana: Implications relating to therapeutic benefits and molecular docking. Food Funct..

[19] Prieto-Blanco M.C., López-Mahía P., Prada-Rodríguez D. (2009). Analysis of residual products in benzyl chloride used for the industrial synthesis of quaternary compounds by liquid chromatography with diode-array detection. J. Chromat. Sci..

[20] Sanad M.H., Gehan M., Saleh F.A. (2017). Marzook. Radioiodination and biological evaluation of nizatidine as a new highly selective radiotracer for peptic ulcer disorder detection. J. Label. Comp. Radiopharmc..

[21] Idan S.A., Al-Marzoqi A.H., Hameed I.H. (2015). Spectral analysis and anti-bacterial activity of methanolic fruit extract of Citrullus colocynthis using gas chromatography-mass spectrometry. Afr. J. Biotechnol..

[22] Lebda M.A., Mostafa R.E., Taha N.M., Abd El-Maksoud E.M., Tohamy H.G., Al Jaouni S.K., El-Far A.H., Elfeky M.S. (2021). Commiphora myrrh supplementation protects and cures ethanol-induced oxidative alterations of gastric ulceration in rats. Antioxidants.

[23] Ibrahim N.K. (2013). Possible protective effect of kombucha tea ferment on cadmium chloride induced liver and kidney damage in irradiated rats. Int. J. Biol. Sci..

[24] Horiguchi H., Oguma E., Kayama F. (2011). Cadmium induces anemia through interdependent progress of hemolysis, body iron accumulation, and insufficient erythropoietin production in rats. Toxicol. Sci..

[25] Andjelkovic M., Buha Djordjevic A., Antonijevic E., Antonijevic B., Stanic M., Kotur-Stevuljevic J., Spasojevic-Kalimanovska V., Jovanovic M., Boricic N., Wallace D. (2019). Toxic effect of acute cadmium and lead exposure in rat blood, liver, and kidney. Int. J. Env. Res. Public Health.

[26] Kacar Kocak M., Yazihan N., Akcil E., Bay M., Aslan O. (2010). The effect of chronic cadmium toxicity on blood pressure and plasma viscosity. Pathophysiol. Haemos Throm..

[27] Kataranovski M., Mirkov I., Belij S., Nikolic M., Zolotarevski L., Ciric D., Kataranovski D. (2009). Lungs: Remote inflammatory target of systemic cadmium administration in rats. Environ. Toxicol. Pharmacol..

[28] Onyenekwe P.C., Odeh C., Nweze C.C. (2012). Volatile constituents of Ogiri, soybean daddawa and locust bean daddawa three fermented Nigerian food flavour enhancers. Electron. J. Environ. Agricul. Food Chem..

[29] Ramu R., Shirahatti P.S., Anilakumar K.R., Nayakavadi S., Zameer F., Dhananjaya B.L., Nagendra Prasad M.N. (2017). Assessment of nutritional quality and global antioxidant response of banana (*Musa* sp.*CV. nanjangud rasa bale*) pseudostem and flower. Pharmacog. Res..

[30] Abbas K., Rizwani G.H., Zahid H., Qadir M.I. (2017). Evaluation of nephroprotective activity of *Musa paradisiaca* L. in gentamicin-induced nephrotoxicity. Pak. J. Pharm. Sci..

[31] Alhazzi I. (2008). Cadmium induced hepatotoxicity and oxidative stress in rats: Protection by selenium. Res. J. Environ. Sci..

[32] Adewoye E.O., Taiwo V.O., Olayioye F.A. (2009). Antioxidant and anti-hyperglycemic activities of *Musa sapientum* root extracts in alloxan-induced diabetic rats. Afr. J. Med. Med Sci..

[33] Panigrahi P.N., Dey S., Sahoo M., Dan A. (2017). Antiurolithiatic and antioxidant efficacy of *Musa paradisiaca* pseudostem on ethylene glycol-induced nephrolithiasis in rat. Indian J. Pharmacol..

[34] Adikwu E., Deo O., Geoffrey O. (2013). Hepatotoxicity of cadmium and roles of mitigating agents. Br. J. Pharmacol. Toxicol..

[35] Renugadevi J., Prabu S.M. (2010). Cadmium-induced hepatotoxicity in rats and the protective effect of naringenin. Experim. Toxicol. Pathol..

[36] Albasha M.O., Azab S. (2014). Effect of cadmium on the liver and amelioration by aqueous extracts of fenugreek seeds, rosemary, and cinnamon in Guinea pigs: Histological and biochemical study. Cell Biol..

[37] Bertin G., Averbeck D. (2006). Cadmium: Cellular effects, modifications of biomolecules, modulation of DNA repair and genotoxic consequences. Biochimie.

[38] Almeer R.S., AlBasher G.I., Alarifi S., Alkahtani S., Ali D., Abdel Moneim A.E. (2019). Royal jelly attenuates cadmium-induced nephrotoxicity in male mice. Sci. Rep. Nat..

[39] Iweala E.E., Oludare F.D. (2011). Hypoglycemic effect, biochemical and histological changes of Spondias mombin Linn. and Parinari Polyandra Benth. Seeds ethanolic extracts in alloxan-induced diabetic rats. Pharmacol. Toxicol..

[40] Verma P., Paswan S.K., Raj A., Nath V., Gupta R., Verma S., Srivastava S., Venketshwara Rao C. (2017). Hematological, antioxidant and protective performance of *Usnea longissima* on chemical induced hepatotoxicity in experimental animals. Coast. Life Med..

[41] Singleton V.L., Orthofer R., Lamuela-Raventós R.M. (1999). Analysis of total phenols and other oxidation substrates and antioxidants by means of folin-ciocalteu reagent. Methods Enzymol..

[42] Zhishen J., Mengcheng T., Jianming W. (1999). The determination of flavonoid contents in mulberry and their scavenging effects on superoxide radicals. Food Chem..

[43] Prieto P., Pineda M., Aguilar M. (1999). Spectrophotometric quantitation of antioxidant capacity through the formation of a phosphomolybdenum complex: Specific application to the determination of vitamin E. Anal. Biochem..

[44] Blois M.S. (1985). Antioxidant determinations by the use of a stable free radical. Nature.

[45] Ebrahimzadeh H. (1998). A revised spectrophotometric method for determination of triterpenoid saponins. Indian Drugs.

[46] Ebrahimzadeh M.A., Pourmorad F., Bekhradnia A.R. (2008). Iron chelating activity, phenol and flavonoid content of some medicinal plants from Iran. Afr. J. Biotechnol..

